# Ubiquitin conjugation to Gag is essential for ESCRT-mediated HIV-1 budding

**DOI:** 10.1186/1742-4690-10-79

**Published:** 2013-07-29

**Authors:** Paola Sette, Kunio Nagashima, Robert C Piper, Fadila Bouamr

**Affiliations:** 1 Viral budding Unit, Laboratory of Molecular Microbiology, National Institute of Allergy and Infectious Diseases, National Institutes of Health, 4 Center Dr, Bethesda, MD 20894, USA; 2 Image Analysis Laboratory, Advanced Technology Program, SAIC-Frederick, NCI-Frederick, Frederick, MD 21702, USA; 3 Molecular Physiology and Biophysics, University of Iowa, Iowa City, Iowa 52246, USA

**Keywords:** Ubiquitin, HIV budding, ESCRT, Deubiquitination, Gag

## Abstract

**Background:**

HIV-1 relies on the host ESCRTs for release from cells. HIV-1 Gag engages ESCRTs by directly binding TSG101 or Alix. ESCRTs also sort ubiquitinated membrane proteins through endosomes to facilitate their lysosomal degradation. The ability of ESCRTs to recognize and process ubiquitinated proteins suggests that ESCRT-dependent viral release may also be controlled by ubiquitination. Although both Gag and ESCRTs undergo some level of ubiquitination, definitive demonstration that ubiquitin is required for viral release is lacking. Here we suppress ubiquitination at viral budding sites by fusing the catalytic domain of the Herpes Simplex UL36 deubiquitinating enzyme (DUb) onto TSG101, Alix, or Gag.

**Results:**

Expressing DUb-TSG101 suppressed Alix-independent HIV-1 release and viral particles remained tethered to the cell surface. DUb-TSG101 had no effect on budding of MoMLV or EIAV, two retroviruses that rely on the ESCRT machinery for exit. Alix-dependent virus release such as EIAV’s, and HIV-1 lacking access to TSG101, was instead dramatically blocked by co-expressing DUb-Alix. Finally, Gag-DUb was unable to support virus release and dominantly interfered with release of wild type HIV-1. Fusion of UL36 did not effect interactions with Alix, TSG101, or Gag and all of the inhibitory effects of UL36 fusion were abolished when its catalytic activity was ablated. Accordingly, Alix, TSG101 and Gag fused to inactive UL36 functionally replaced their unfused counterparts. Interestingly, coexpression of the Nedd4-2s ubiquitin ligase suppressed the ability of DUb-TSG101 to inhibit HIV-1 release while also restoring detectable Gag ubiquitination at the membrane. Similarly, incorporation of Gag-Ub fusion proteins into virions lifted DUb-ESCRT inhibitory effect. In contrast, Nedd4-2s did not suppress the inhibition mediated by Gag-DUb despite restoring robust ubiquitination of TSG101/ESCRT-I at virus budding sites.

**Conclusions:**

These studies demonstrate a necessary and natural role for ubiquitin in ESCRT-dependent viral release and indicate a critical role for ubiquitination of Gag rather than ubiquitination of ESCRTs themselves.

## Background

Most enveloped viruses need to traverse the cell membrane to acquire their membrane and separate to spread infection, a process that involves the function of the ESCRT apparatus. ESCRT members facilitate membrane fission events necessary for budding of vesicles into multivesicular bodies (MVB) [1] and abscission of daughter cells at the completion of cytokinesis [2]. These processes share a common topology with virus budding in that they all involve membrane scission, which severs membranous necks away from the cytoplasm [3,4]. The ESCRT pathway is comprised of four multi-protein complexes, named ESCRT-0, I, II and III. An ordered recruitment of ESCRT-I and a subset of ESCRT-III components are believed to facilitate membrane-scission necessary for virus budding [5,6]. The latter and ESCRT recycling requires the activity of the AAA-ATPase VPS4 [7].

Gag proteins carry highly conserved sequences called Late or L domains to recruit ESCRT components to sites of virus budding. Three types of L domains have been identified so far and carry the PTAP, LYPXnL and PPXY sequences, which bind Tsg101, Alix and members of the Nedd4-like ligase family, respectively. Tsg101 functions as part of ESCRT-I [8] and requires specific isoforms of ESCRT-III to promote HIV budding [5], mechanisms of recruitment however are not known. In contrast Alix binds directly ESCRT-III members, the Charged Multivesicular Proteins CHMP4 isoforms to sever HIV away from cells [9,10]. The reliance of the PPPY/Nedd4-like pathway on ESCRT-III function has been demonstrated [11-13] although factors bridging interactions between Nedd4-like ligases and ESCRT-III are not known. Nedd4-like Ub ligase loss of enzymatic activity correlated with inability to function in virus release revealing the importance for ubiquitin (Ub) conjugation to components of virus budding sites. Early studies suggested that Ub also plays a role in HIV budding [14-16]. Interestingly, despite the absence in Gag of canonical sequences that mediate interactions with Nedd4-like ligases, HIV-1 is sensitive to their stimulatory effects [17-19].

At the endosome, ESCRT-0, I, II and III are believed to act collectively and concertedly to sort ubiquitinated cargo proteins into MVB [1,20]. Indeed, covalent Ub conjugation is necessary and sufficient for the entry of cargo into the degradative MVB/lysosomal pathway [21]. The ESCRT pathway is necessary for trafficking cell surface membrane proteins into MVBs, supporting the notion that conjugating Ub to cargo serves as a signal for ESCRT-dependent entry and sorting in MVB compartments [22]. Evidence of a role for Ub in ESCRT-mediated virus budding is supported by several findings [17,23-27], although a direct role for such function has not been shown.

HIV-1 Gag is ubiquitinated near NC and p6 regions [28] and preventing Ub conjugation to Gag or following cumulative mutation of lysine residues inhibited virus release [29]. Both free Ub and ubiquitinated Gag molecules are found in virions [30] and Gag ubiquitination is highly dependent on its association with the plasma membrane [31] implying functional significance for Ub in late assembly events. Other studies suggested that in a certain context Gag ubiquitination might be dispensable for virus budding [32]. Alleviation of HIV budding defects by deposition of Ub in the vicinity of Gag [26,27] suggested a role for ubiquitination of Gag-binding proteins in HIV budding.

Both TSG101 and Alix are ubiquitinated [18,33] and bind Ub themselves [23,34-36]. Alix loss of Ub binding sites suppressed function in virus release [37]. Interferon-induced inhibition of Gag ubiquitination interfered with TSG101 recruitment [24] suggested a role for Ub-conjugation to Gag in virus release *in vivo*. Appending Gag with a Ub molecule alleviated virus budding defects due to lack of access to the ESCRT pathway suggesting that Ub fusion to Gag substitutes for the absence of L domain function(s) [23,25]. Similarly, covalent conjugation of Ub to Gag correlated with ESCRT-dependent virus budding [12], implying ubiquitination of Gag might be involved in the recruitment/utilization of ESCRT members. A natural and necessary role for Ub conjugation to either component of particle assembly sites in virus budding is yet to be shown.

In this study, we generated ubiquitination-resistant virus assembly sites to investigate the role of Ub in virus budding. Gag, TSG101 and Alix became ubiquitination-resistant following fusion with the Herpes Virus UL36 catalytic domain (DUb). DUb fusion proteins retained their known protein-protein interactions and efficiently inhibited virus budding in a DUb enzymatic activity-dependent manner. DUb inhibitory effects were alleviated and virus release restored upon incorporation of Ub molecules into sites of budding as Gag-Ub fusion proteins. In absence of ESCRTs ubiquitination, Gag ubiquitination at the membrane was sufficient to mediate virus production. Conversely, budding defects due to Gag deubiquitination were irreversible despite Gag-association with detectably hyperubiquitinated ESCRT complexes at the membrane. Our data provide direct demonstration that Ub plays an important and necessary role in ESCRT-mediated viral budding and indicate that it is ubiquitination of Gag itself that plays the critical role.

## Results

Multiple lines of evidence implicate a role for ubiquitin (Ub) in the process of viral budding and release [14-17,23,26,27,29]. For instance, both ESCRTs and Gag undergo some level of ubiquitination. Experimental manipulations such as overexpression of Ub ligases that increase ubiquitination of Gag and Gag-associated proteins promote viral release [17,18,26,27] and fusion of Ub directly to Gag can promote fusion and circumvent requirement for directly binding ESCRT components via L domain sequences [23]. Despite these indications, two fundamental questions remain regarding the potential role for Ub in ESCRT-dependent viral release. While most of these studies establish that Ub can be sufficient to mediate viral budding, it has yet to be shown that Ub provides a natural and necessary role in the process. Rather, the presence of Ub in these experimental settings could simply represent an artificial means of recruiting ESCRT-components that are known to harbor multiple Ub-binding domains. The other issue is establishing what specific protein(s) in the viral budding process requires ubiquitination given that either ubiquitintion of Gag or some other non-Gag protein can be sufficient for release [12,26,27]. In order to directly assess the role of Ub in HIV-1 budding, we used a method recently described by [38] which relies on fusing the catalytic domain of a deubiquitinating enzyme (DUb) onto a protein of interest. This approach blocks the ability of that protein, as well as other tightly-associated interacting proteins, from accumulating in a ubiquitinated form. This simple approach to converting a given protein into an ubiquitination-resistant form provides a complementary control in the form of the same fusion but with an inactivating Cys-Ser mutation in the catalytic site of the DUb. To exploit this approach, we expressed DUb fusion proteins to Alix, TSG101 and HIV Gag proteins and assessed the effects on virus release.

### Effect of DUb-TSG101 on viral release

**Figure 1:**
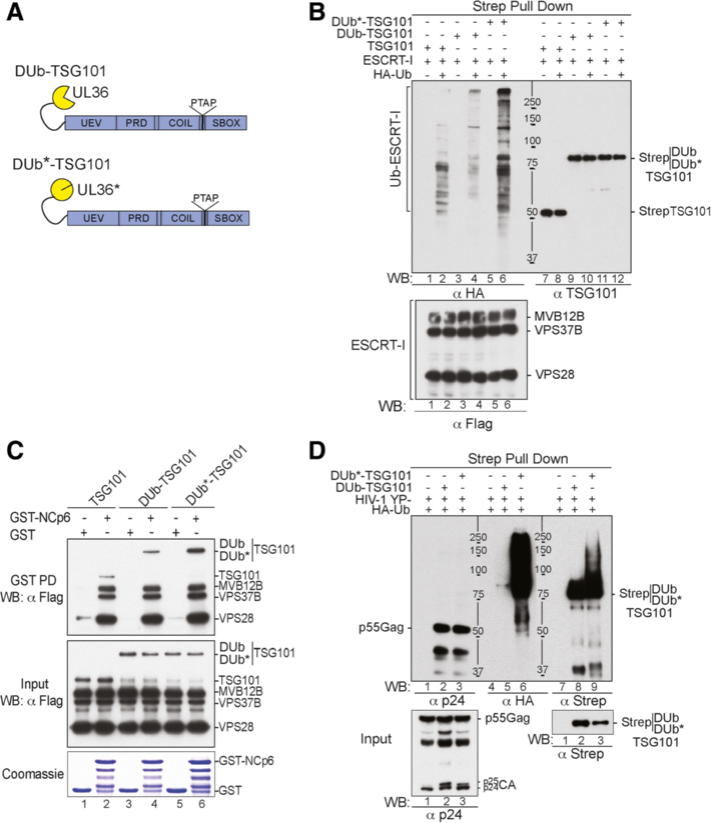
**Fusion of the UL36 catalytic domain DUb to TSG101 inhibits ESCRT-I ubiquitination. (A)** Schematic representation of the DUb-TSG101 fusion proteins. DUb and DUb* domains were fused to TSG101 as depicted. **(B)** Effect of DUb fusion to TSG101 on ESCRT-I ubiquitination. 293T cells were transfected with ESCRT-I members [Flag-tagged VPS28 (800 ng), VPS37 (1.7 μg) and MVB12 (800 ng)] and either strep-TSG101 (2.5 μg) (lanes 1 and 7), DUb-TSG101 (lanes 3 and 9) or DUb*-TSG101 alone (600 ng) (lanes 5 and 11) or with HA-Ub (1.5 μg throughout the study unless otherwise specified) (lanes 2, 4, 6, 8, 10, 12). Immunocomplexes were analyzed by western (WB) blotting (WB) using the indicated antibodies. **(C)** DUb-TSG101 fusion proteins bind HIV-1 NCp6 region. GST (lanes 1, 3 and 5) and GST-NCp6 (lanes 2, 4 and 6) were captured on beads and then incubated with lysates from 293T cells expressing Flag-ESCRT-I/Tsg101 (lane 2), DUb-TSG101 (lane 4) or DUb*-TSG101 (lane 6). Captured proteins and cell lysates were analyzed by WB using the anti-Flag antibody. GST fusion proteins were visualized by Coomassie blue staining. **(D)** DUb-TSG101 deubiquitinated Gag assembly complexes. 293T cells were co-transfected with HIV-1 YP- mutant (1 μg throughout the study unless otherwise specified) and HA-Ub alone (lanes 1, 4 and 7), with strep-DUb-TSG-101 (lanes 2, 4 and 6) or strep- DUb*-TSG-101 (lanes 3, 6 and 9). Insoluble Gag-enriched fractions were isolated and solubilized to *Strep-Tactin*-capture DUb-TSG101 or DUb*-TSG101 containing complexes. Gag proteins associated with these complexes and their ubiquitination status assessed by antibodies to p24 and HA, respectively, and input fractions were probed with the indicated antibodies.

The ESCRT-I component TSG101 binds directly to PTAP motifs within the HIV-1 Gag protein and eliminating the PTAP motif or the ability of TSG101 to bind Gag reduces viral release by ~80%. TSG101 can also non-covalently bind Ub via its UEV domain and also undergoes ubiquitination [39]. We first constructed a DUb fusion to TSG101 by creating a protein comprised of the catalytic domain from the Herpes Simplex UL36 DUb fused onto the N-terminus of TSG101 (Figure 1A). The DUb-TSG101 also contained an N-terminal bivalent Strep tag for affinity purification. As a complementary control, we also constructed a version TSG101 fused to UL36 that was catalytically inactivated by a single point mutation (DUb*). Active and inactive DUb-TSG101 and DUb*-TSG101, respectively, were co-expressed in 293T cells along with HA-Ub and affinity isolated from cell lysates on *Strep*-tactin beads (Figure 1B, upper). Complexes with inactive DUb*-TSG101 showed high levels of ubiquitinated proteins revealed by anti-HA western blot. In contrast, the active DUb-TSG101 fusion had dramatically less ubiquitinated proteins (compare lanes 4–6) even though the levels of DUb-TSG101 and DUb*-TSG101 were comparable (compare lanes 10 and 12). Affinity-isolated TSG101, DUb-TSG101 and DUb*-TSG101 also showed similar levels of association with the other ESCRT-I components MVB12B, VPS37B and VPS28 indicating that the fusion of active or inactive UL36 catalytic domain did not interfere with the ability of TSG101 to assemble into its native ESCRT-I complex. (Figure 1B lower, compare lanes 1, 3 and 5).

Lysates from cells expressing Flag-tagged TSG101, DUb-TSG101, or DUb*-TSG101 in combination with the other Flag-tagged ESCRT-I subunits were also subjected to GST-pulldown assays with GST alone or GST-fused to a fragment encompassing the NC and p6 regions of the HIV-1 Gag protein that contains binding sites for Alix and the PTAP binding site for TSG101. Figure 1C shows that GST-NCp6 captured ESCRT-I containing either TSG101, DUb-TSG101, or DUb*-TSG101 comparably. Interestingly, while the levels of GST-NCp6 bound MVB12B, VPS37B, and VPS28 were more similar, a greater proportion of DUb*-TSG101 was bound by GST-NCp6 than WT TSG101 or active DUb-TSG101. Together, these data show that both active and inactive DUb-TSG101 and DUb*-TSG101 retain their ability to assemble into ESCRT-I complexes and interact with HIV-1 Gag proteins.

To examine the effect of DUb-TSG101 on Gag assembling complexes, we captured DUb-TSG101 recruited at the membrane by nascent virus particles and checked their ubiquitination status. DUb-TSG101 and DUb*-TSG101 were affinity captured on *Strep*-tactin beads from cells expressing HA-Ub and co-expressing HIV-1YP-. The captured TSG101-containing complexes were immunoprecipitated from cell fractions known to be enriched in membrane-associated insoluble Gag assembling proteins [31]. Immunocomplexes were immuno-blotted for Gag using anti-p24 antibodies (Figure 1D, lanes 1–3), levels of ubiquitinated proteins using an anti-HA antibody (Figure 1D, lanes 4–6), and for the TSG101 protein itself using an anti-Strep antibody (Figure 1D, lanes 7–9). Complexes containing DUb-TSG101 had greatly diminished levels of ubiquitinated proteins compared with complexes of inactive DUb*-TSG101 (lanes 5 and 6). No high molecular weight forms of Gag were observed in the anti-p24 (lanes 2 and 3) or the anti-HA immunoblots when Gag was co-expressed with DUb-TSG101 in contrast to complexes of DUb*-TSG101 (compare lane 5 to 6 and lane 8 to 9). These data indicate that direct association with DUb-TSG101 generated ubiquitination-resistent TSG101/ESCRT complex and protected Gag complexes against ubiquitination.

**Figure 2:**
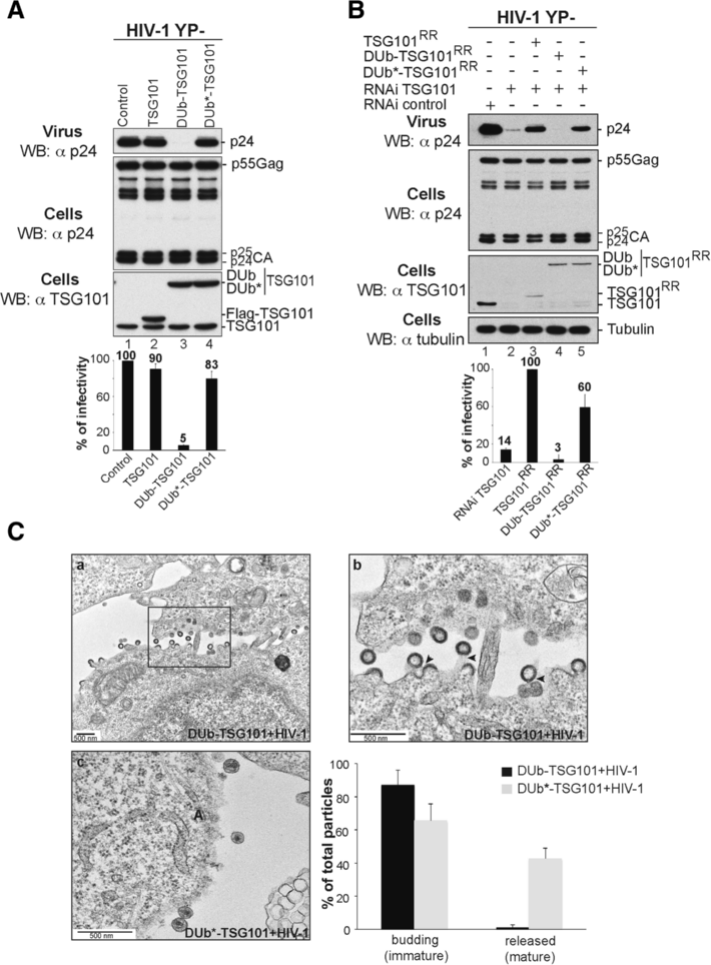
**DUb-TSG101 interferes with HIV-1 release. (A)** Co-expression of DUb-TSG101 inhibits HIV-1 release. 293T cells were transfected with expression plasmids of HIV-1 YP- (lane 1), or co-expressing Flag-TSG101, Flag-DUb-TSG101 or Flag-DUb*-TSG101 (lanes 2, 3, 4, respectively). **(B)** DUb-TSG101 failed to replace functionally cellular TSG101. 293T cells were transfected twice with RNAi to TSG101 (lanes 2–5) at 36-h intervals. At the second transfection, cells were co-transfected with expression plasmids of HIV-1 YP- alone (lanes 1 and 2) or either Flag-TSG101^RR^ (250 ng) (RNAi Resistant form), Flag-DUB-TSG101 ^RR^ or Flag-DUb*-TSG101^RR^ (15 ng) (lanes 3, 4, 5, respectively). Cells and viruses were collected 24 hours post-transfection and their protein content was analyzed by WB using the indicated antibodies. Virus release efficiency was also quantified using HeLa TZM-bl assays from 3 independent experiments and expressed relative to WT HIV **(A)** or WT TSG101 ^RR^**(B)**. **(C)** DUb-TSG101 inhibits late steps of HIV-1 budding. Shown are EM images of thin-sectioned 293T cells co-transfected with HIV-1 and DUb-TSG101 **(a and b)** or with DUb*-TSG101 **(c).** A high-magnification image of budding virus particles from panel **(a)** (rectangle) is shown **(b)** and black arrows indicate particles tethered to the plasma membrane or to each other. Quantification of budding defects was performed and approximately >250 virus particles from 2 independent experiments were examined and categorized as immature budding particles, or mature released particles (±SD).

We next assessed the impact of fusing active and inactive DUb onto TSG101 for HIV-1 release since TSG101 and other ESCRT-I subunits are required for efficient scission of HIV-1 from the cell surface [8]. Expressing DUb-TSG101 inhibited Gag processing as demonstrated by the accumulation of the CA-p1 (p25CA form) and caused a 70% reduction in virus release (Additional file 1: Figure S1A, lane 3). The remaining 30% could either be due to incomplete compromise of endogenous TSG101 function or instead be due to the alternative use of Alix, which is mediated by a LYPXnL motif within the HIV-1 Gag protein that mediates direct binding to Alix and has been shown to be sufficient for viral release. To distinguish between these possibilities, we assessed the impact of DUb-TSG101 on the release of mutant HIV-1 that lacks its Alix-binding motif (HIV-1 YP-). DUb-TSG101 blocked over 95% of release of HIV-1 YP- (Figure 2A, lane 3). These data suggest that the inhibitory effects of DUb-TSG101 on HIV-1 release are specific for the interaction between HIV Gag and TSG101. As further demonstration of this specificity, we found that DUb-TSG101 had no effect on release of MoMLV or EIAV, which use TSG101-independent pathways for budding (Additional file 1: Figure S1B and S1C). Furthermore, a mutant DUb-TSG101 that lost the ability to bind HIV Gag (M95A) had no effect on HIV budding (Additional file 1: Figure S1D, lane 3) demonstrating that DUb-TSG101 inhibitory effect requires specific recruitment to Gag assembly sites. No effect on viral release of HIV-1, HIV-1 YP-, MoMLV or EIAV was found upon expressing inactive DUb*-TSG101 at comparable levels suggesting that the inhibitory effect of DUb-TSG101 was due to the enzymatic activity of the fused UL36-DUb. Remarkably, Flag-tagged DUb*-TSG101 could functionally replace endogenous TSG101 depleted by RNAi just as well as WT Flag-tagged TSG101 in facilitating the release of HIV-1 (Figure 2B, lanes 3 and 5). As expected, enzymatically active DUb-TSG101 failed to drive release of virus when endogenous TSG101 was depleted (Figure 2B, lane 4). These data further demonstrate the specificity of the inhibitory effect of DUb-TSG101, indicating that it is mediated by deubiquitination rather than mere fusion of a globular domain to the TSG101 N-terminus.

To better characterize how DUb-TSG101 inhibited HIV-1 release, we examined cells co-expressing HIV-1 and DUb-TSG101 by transmission electron microscopy (Figure 2C). Cells expressing DUb-TSG101 showed multiple viral particles that were tethered to each other or to the cell surface with elongated budding necks, indicating a severe defect in membrane scission (Figure 2C panels a and b). In contrast, cells expressing inactive DUb*-TSG101 were able to produce mature HIV-1 particles that were clearly detached from the cell surface (Figure 2C, panels c). These data were confirmed in duplicate experiments and quantified by counting >200 viral particles within multiple fields (Figure 2C). Taken together these results indicated that deubiquitination of TSG101 prevented HIV-1 budding and separation from cells. This effect is similar to that observed when viral Gag proteins are unable to engage an active ESCRT apparatus.

### Effect of DUb-Alix on viral release

**Figure 3:**
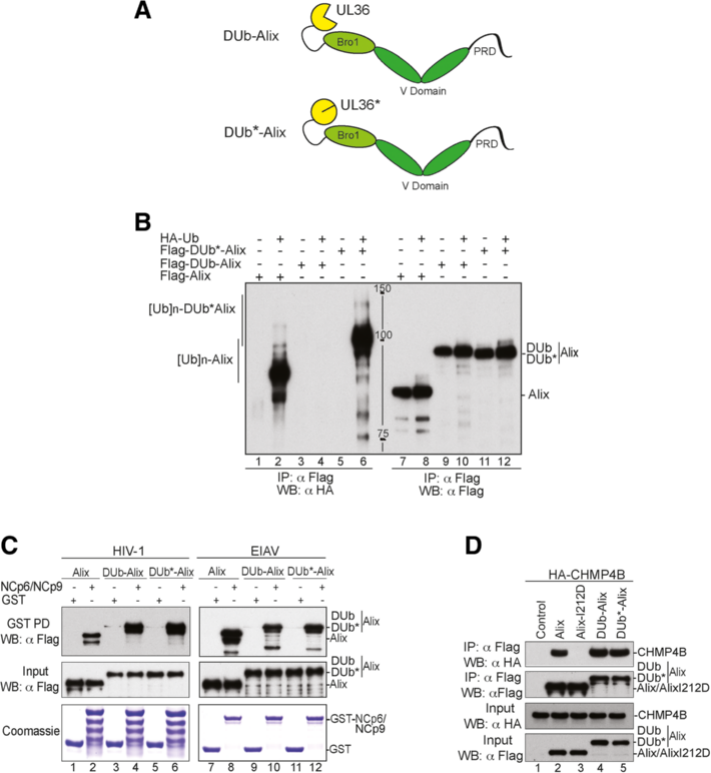
**Fusion with DUb had no effect on Alix known protein-protein interactions. (A)** Schematic representation of the DUb-Alix fusion proteins. The active or inactive UL36 DUb catalytic domain was fused to Alix N-terminal region as depicted. **(B)** Effect of DUb fusion on Alix ubiquitination. 293T cells were transfected with Flag-Alix (500 ng), Flag-DUb-Alix (500 ng) or Flag-DUb*-Alix (500 ng) (lanes 1, 3, 5, 7, 9 and 11; respectively) or in combination with HA- Ub (lines 2, 4, 6, 8, 10 and 12; respectively). Immunocomplexes were analyzed by WB blot using the indicated antibodies. **(C)** DUb-Alix fusion proteins bind HIV-1 NCp6 and EIAV NCp9 proteins. GST, GST-NCp6 (right panel) or GST-NCp9 (left panel) fusion proteins were purified on glutathione beads and then incubated with lysates from 293T cells expressing 1.5 μg of Flag-Alix (lanes 2 and 8), Flag-DUb-Alix (lanes 4 and 10) or Flag-DUb*-Alix (lanes 6 and 12). Captured proteins and cell lysates were analyzed by WB blot using an anti-Flag antibody and GST fusion proteins visualized by Coomassie blue staining. **(D)** DUb-Alix fusion proteins retain binding to CHMP4B. 293T cells were co-transfected with HA-CHMP4B alone (2 μg) (control), or in combination with 1 μg of Flag-Alix (lane 2), Flag-AlixI212D (lane 3), Flag-DUb-Alix (lane 4) or Flag-DUb*-Alix (lane 5). Cell lysates were incubated with anti-Flag antibody-conjugated beads and both input and immunocomplexes analyzed by WB blot using the indicated antibodies.

Several viral Gag proteins directly bind ESCRT-associated Alix as a means to interact with the ESCRT apparatus to induce viral budding and release. Like TSG101, Alix undergoes ubiquitination and also binds Ub suggesting its function or regulation is Ub-dependent. However the functional significance of these properties in virus budding is not known. To determine whether there was a role for Ub in Alix-dependent viral budding, we assessed the effect of expressing DUb-Alix and DUb*-Alix comprised of Alix fused to the C-terminus of active or inactive UL36 DUb, respectively. These proteins were also tagged with the Flag epitope (Figure 3A). When co-expressed in 293T cells with HA-Ub and immunoprecipitated with anti-Flag antibodies, WT Flag-Alix as well as DUb*-Alix were readily found to be ubiquitinated as revealed by immunoblotting samples with anti-HA. In stark contrast, Ub conjugates were completely absent from immunoprecipitates of active DUb-Alix thus demonstrating the effectiveness of the DUb domain in eliminating ubiquitination from Alix (Figure 3B). Both active and inactive DUb-Alix and DUb*-Alix retained their ability to interact GST-fusion proteins encompassing the NC and p6 late domain region of HIV-1 or the NC and p9 late domain of EIAV, both of which are known to interact with Alix and which are capable of using an Alix-dependent pathway for viral budding (Figure 3C). In addition, immunoprecipitation experiments (Figure 3D) demonstrated that DUb-Alix and DUb*-Alix retained their ability to interact with CHMP4B. Immunoprecipitation experiments also showed that both DUb-Alix and DUb*-Alix could oligomerize with full-length WT Alix or a fragment of Alix containing the middle V domain and the proline-rich C-terminal tail (Additional file 1: Figure S2). Together, these data demonstrate the fusion of the UL36 DUb domain to the N-terminus of Alix did not interfere with its ability to interact with its known-binding partners.

**Figure 4:**
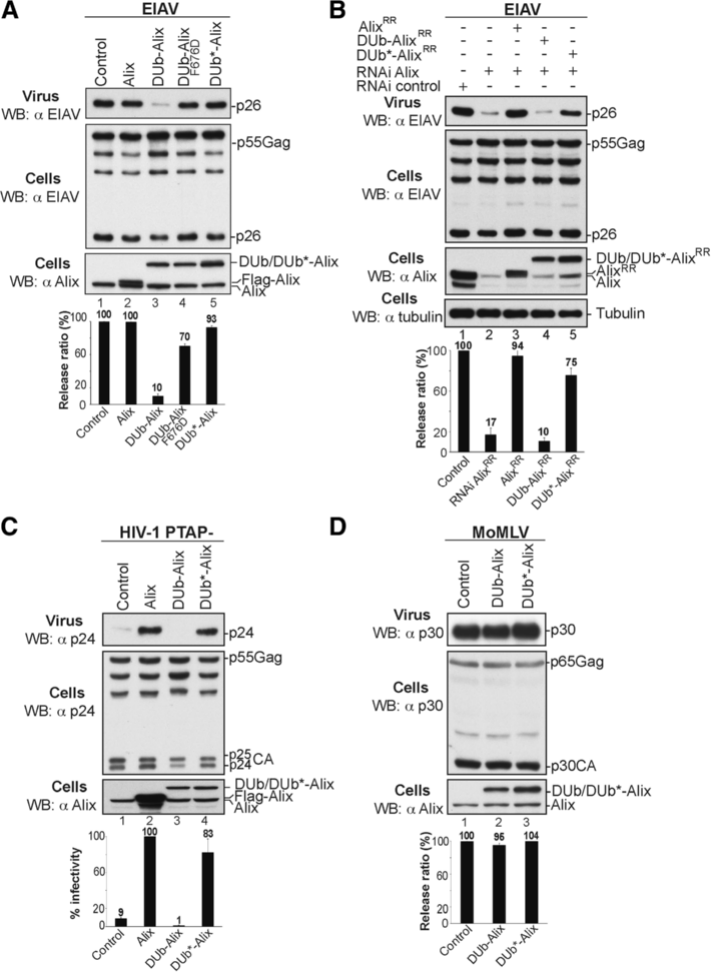
**DUb-Alix interferes with Alix mediated virus release. (A)** Co-expression of DUb-Alix inhibits EIAV release. 293T cells were transfected with EIAV proviral DNA alone (500 ng throughout the study unless otherwise specified) (lane 1), with Flag-Alix, Flag-DUb-Alix, Flag-DUb-AlixF676D or Flag-DUb*-Alix (lanes 2, 3, 4, 5; respectively). **(B)** The active DUb-Alix fusion protein failed to replace cellular Alix to promote EIAV release. 293T cells were transfected twice with Alix RNAi oligonucleotides at 36-h intervals. At the second transfection, cells were transfected with EIAV provirus alone (lanes 1 and 2) or with 100 ng of RR versions of Flag-Alix, Flag-DUb-Alix or Flag-DUb*-Alix (lanes 3, 4, 5; respectively). **(C)** DUb-Alix fails to rescue HIV-1 PTAP- budding. 293T cells were transfected with expression plasmids of HIV-1 PTAP- alone (1 μg) (lane 1), or in combination with Flag-Alix (500 ng), Flag-DUb-Alix (100 ng) or Flag-DUb*-Alix (100 ng) (lanes 2, 3, 4; respectively). **(D)** Co-expression of DUb-Alix has no effect on MoMLV release. 293T cells were transfected with MoMLV provirus alone (1 μg) (lane 1), with Flag-DUb-Alix (100 ng) (lane 2) or Flag-DUb*-Alix (100 ng) (lane 3). Protein content of pelleted virions and cell lysates was analyzed 24 h later by WB blotting with the indicated antibodies. Release ratio (%) was calculated using the formula described in supplemental material. Error bars represent standard deviations (SD).

We next assessed whether DUb-Alix had an effect on the release of two viruses that require Alix for their budding and release. These were EIAV and a mutant HIV-1 (HIV PTAP-) where the PTAP motif within its Gag protein that mediates binding to TSG101 was eliminated (Figure 4). We found that expressing DUb-Alix dramatically inhibited EIAV virus production by 90%. This effect required the interaction of EIAV Gag with DUb-Alix since this inhibitory effect was suppressed when the EIAV binding site within the V domain of Alix was compromised by a F676D point mutation [9,40] (Figure 4A, lane 4). The level of specificity demonstrated by the DUb-Alix F676D mutant is important since it indicates that the inhibitory effect of DUb-Alix is not due to recruiting DUb activity to CHMP4B or to other proteins that associate with other regions of Alix. Specificity was also demonstrated by the observation that inactive DUb*-Alix expressed to the same levels of active DUb-Alix had no effect on release of EIAV (Figure 4A, lane 5). In addition, DUb*-Alix, but not the active DUb-Alix, was able to restore release of EIAV from cells depleted of endogenous Alix by RNAi (Figure 4B, lane 5).

DUb-Alix had a similar inhibitory effect on budding of HIV-1 PTAP- virus. Normally, production of HIV-1 PTAP- virus is low in 293T cells [41,42] but can be greatly stimulated by overexpression of WT Alix. However, not only did DUb-Alix lack the ability to stimulate release of HIV-1 PTAP- virus (Figure 4C), it also inhibited 90% of virus release supported by endogenous levels of Alix (Figure 4C, compare lanes 1 and 3). The inhibitory effect of DUb-Alix on release of HIV-1 PTAP- was eliminated when the DUb activity was inactivated (lane 4). Little effect of DUb-Alix was observed on release of MoMLV (Figure 4D), which exits cells independently of Alix [12]. Similarly Alix caused only a minor (20%) reduction in the release of WT HIV-1, which more readily utilizes a TSG101-dependent release pathway than it does an Alix-dependent pathway (Additional file 1: Figure S3). Together these data demonstrate that the inhibitory effects of DUb-Alix are mediated by the enzyme activity of the fused UL36 DUb and emphasize that inhibition requires DUb-Alix to directly interact with viral Gag protein.

### DUb-Gag co-assembles with HIV-1 nascent particles and inhibits virus budding

We found that fusion of UL36 DUb to TSG101 or Alix dominantly interfered with release of viruses that utilize TSG101 or Alix dependent budding pathways. Release of HIV-1 by DUb-TSG101 was halted specifically at the plasma membrane, which caused the accumulation of arrested budding viral particles with remarkable efficiency. While these data demonstrate a role for Ub in virus release, it remained unclear whether such defects results from deubiquitination of TSG101 or Alix or rather the Gag proteins with which they interact. We reasoned that if Gag ubiquitination is important for virus exit, fusing DUb to Gag should instigate a very powerful block on HIV-1 exit, regardless of the ubiquitination status of TSG101 or Alix.

**Figure 5:**
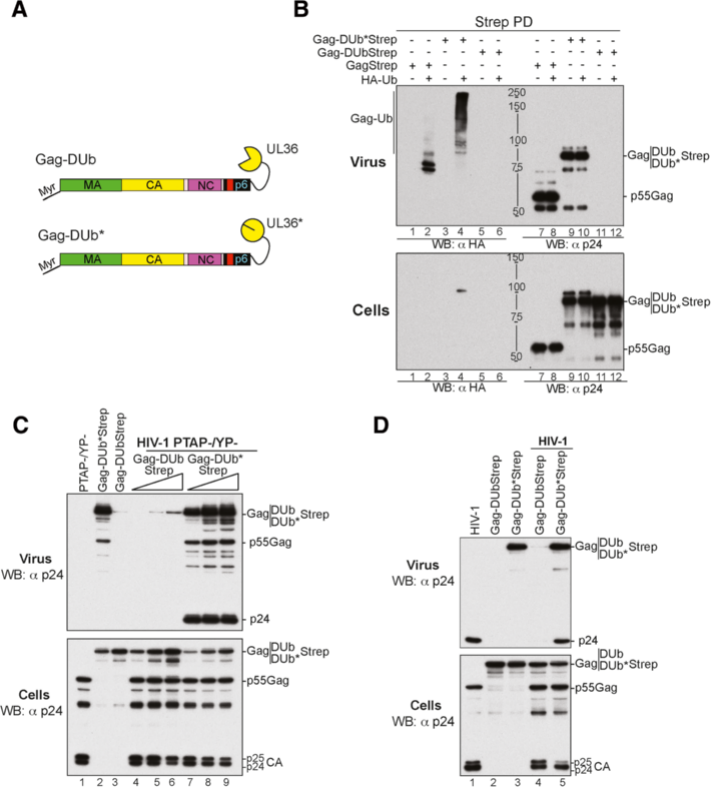
**Fusion to DUb suppresses Gag ubiquitination and ability to release virus. (A)** Schematic representation of DUb-Gag fusion proteins. DUb catalytic domain was fused to Gag C-terminal end. **(B)** DUb fusion to Gag suppresses ubiquitination. 293T cells were transfected with strep-tagged Gag, Gag-DUb or Gag-DUb* fusion proteins (lanes 1, 3, 5, 7, 9, 11; respectively) or in combination with HA-Ub (lanes 2, 4, 6, 8, 10, 12; respectively). Cells and virus pellets were collected 24h post-transfection and incubated with *Strep-Tactin* beads and captured complexes were probed with the indicated antibodies. **(C)** Gag-DUb* fusion protein co-assembled with WT Gag and restored the release HIV-1 PTAP-/YP-. 293T cells were transfected with HIV-1 PTAP-/YP- alone (lane 1), or Gag-DUb*Strep (lane 2), Gag-DUbStrep (lane 3), or with HIV-1 PTAP-/YP- and increasing amounts of either Gag-DUbStrep (500 ng, 1 μg or 2 μg) (lane 4, 5, 6) or Gag-DUb*Strep (500 ng, 1 μg or 2 μg) (lane 7, 8, 9). **(D)** Gag-DUb fusion failed to release virus particles and inhibited HIV-1 release *in trans*. 293T cells were transfected with HIV-1 alone (1 μg) (lane 1), in combination with either Gag-DUbStrep (1.5 μg) (lane 4), or Gag-DUb*Strep (1.5 μg) (lane 5), or with Gag-DUbStrep or Gag-DUB*Strep alone (1.5 μg) (lanes 2, 3; respectively). Cells and virions were harvested as above and their protein content were analyzed by WB blot using an anti-p24 antibody.

**Figure 6:**
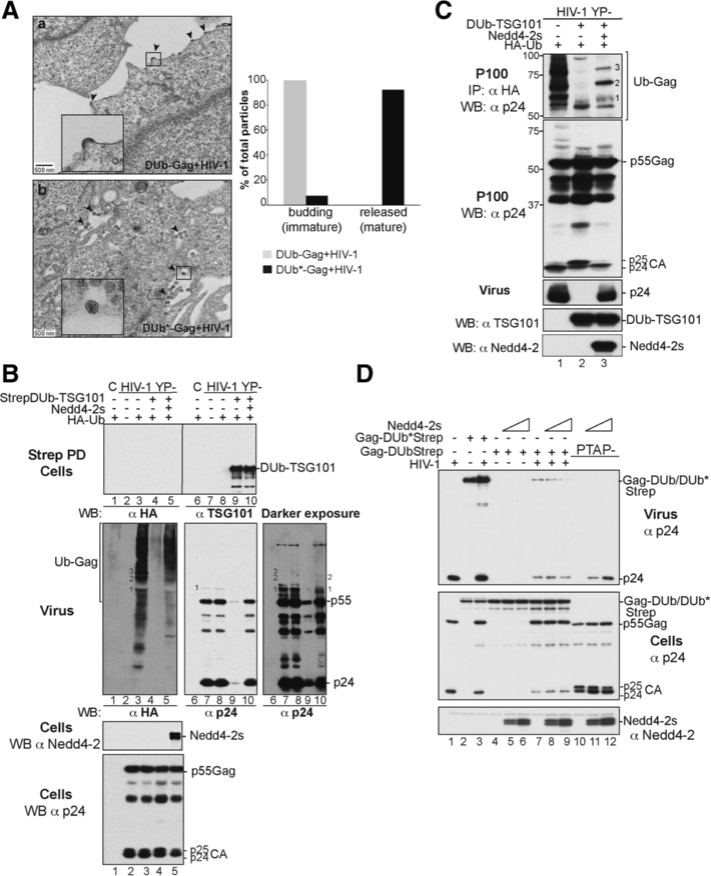
**Nedd4-2s mediated Gag ubiquitination correlates with virus release. (A)** Gag fusion with DUb inhibits virus budding. EM images of thin-sectioned cells co-transfected with HIV-1 and Gag-DUb **(a)** or Gag-DUb* **(b)**. Insets show high-magnification images of a budding or released virus particle and black arrows indicate arrested assembly sites **(a)** or virions **(b)**. Quantification of budding defects was performed as described in Figure 2. **(B)** Nedd4-2s-mediated Gag-ubiquitination relieves DUb-TSG101 inhibitory effect on HIV-1 release. Cells were transfected with HA-Ub (lane 1) or HIV-1 YP- alone (lane 2), or with both alone (lane 3), in combination with Strep-DUb-TSG101 (200 ng) (lanes 4) or with the 3 precedent plasmids and Nedd4-2s (150 ng) (lane 5). Cells and virus were harvested after 24 h; cell lysates were incubated with *Strep-Tactin* beads and the complexes (upper panel), virus pellets (middle panel) and input fractions (lower panels) were probed with the indicated antibodies. **(C)** Nedd4-2s ubiquitinates Gag at the membrane. Cells expressing HIV YP- were co-transfected with either HA-Ub alone (lane 1), with DUb-TSG101 alone (lane 2) or in combination with Nedd4-2s (lane 3). The P100 fractions of each sample were either analyzed by WB using anti-p24CA antibody (second panel from top) or incubated with anti-HA antibody coated beads. Eluates were analyzed by WB with anti-p24CA antibody to detect ubiquitinated Gag molecules. **(D)** Nedd4-2s failed to relieve the inhibitory effect of DUb-Gag. 293T cells were transfected with HIV-1 (1 μg) (lane 1), Gag-DUb*Strep (1.5 μg) (lane 2) or with both (lane 3), with Gag-DUbStrep alone (1.5 μg) (lane 4) or with increasing amounts of Nedd4.2s (100 and 200 ng) (lanes 5 and 6). Cells were also transfected with Gag-DUbStrep in combination with HIV-1 (lane 7) or with increasing amount of Nedd4-2s (lanes 8 and 9). Triangles indicate lanes where increasing amounts of Nedd4-2s are expressed.

We fused enzymatically active and inactive UL36 to the C-terminus of HIV-1 Gag protein that also contained a divalent strep tag (Figure 5A). Normally, expression of HIV-1 Gag results in the production and release of viral-like particles and a portion of the Gag protein contained within them is ubiquitinated. Expression of strep-tagged WT HIV-1 Gag resulted in release of Gag particles from cells (Figure 5B lanes 7 and 8, upper). Similarly, expression of a catalytically dead Gag-DUb* was also efficiently released from cells (lane 9 and 10). In addition, affinity isolation of released Gag from cells showed that both released WT Gag and Gag-DUb* were ubiquitinated (lanes 2 and 4). In contrast, Gag-DUb containing enzymatically active UL36 was not released from cells (lanes 11 and 12,), despite a level of expression comparable to Gag-DUb* (lanes 11 and 12, lower). Additionally, the intracellular Gag-DUb was not ubiquitinated as would be predicted by the presence of the active UL36, whereas a modest signal was detected for Gag-DUb* (Figure 5B, lower). Further experiments showed that Gag-DUb could also dominantly interfere with budding of HIV-1 demonstrating the Gag-DUb inhibits in *trans*. For instance, Gag-DUb* was able to drive release of mutant HIV-1 lacking both Alix and TSG101 binding sites (HIV-1 PTAP-/YP-) (Figure 5C, lanes 7–9) indicating that this Gag fusion protein could co-assemble with the mutant HIV-1 Gag-containing budding virus and provide functional late domain PTAP (TSG101) and LYPXnL (Alix) binding sites for interaction and scission by ESCRTs. In contrast, enzymatically active Gag-DUb did not rescue budding of HIV-1 PTAP-/YP- virus (lanes 4–6). In addition, Gag-DUb also dominantly interfered with the release of WT HIV-1 virus (Figure 5D, lane 4), which retained the ability to interact with cellular TSG101 and Alix. Inhibition of HIV-1 budding by Gag-DUb was greater than 90%, demonstrating that viral particles were unable to use either the Alix-dependent or TSG101-dependent release pathways. Further characterization of Gag-DUb inhibitory effect on virus release revealed arrested budding structures at the plasma membrane when HIV-1 was co-expressed with Gag-DUb (Figure 6A, a). In contrast cells co-expressing Gag-DUb* and HIV displayed almost exclusively mature free virions around them (Figure 6A, b and quantification panel). Together, these data emphasize the idea that Ub plays an important and necessary role in viral budding and indicate that it is ubiquitination of Gag proteins themselves that plays the critical role.

### Ubiquitination of Gag correlates with virus release

To better discern what the important target of ubiquitination was in the process of viral release, we took advantage of previous observations demonstrating a potential role of Nedd4-family Ub-ligases in promoting virus release. A variety of studies have demonstrated that overexpressing Nedd4-2s can compensate for some defects in ESCRTs or viral Gag proteins that otherwise lead to inefficient budding. However, it has always been difficult to determine what the relevant targets of those ubiquitination events are. We assessed whether co-expression of Nedd4-2s [17,19] would have an effect on the ability of DUb-TSG101 to inhibit budding of mutant HIV-1 YP- virus that relies exclusively on TSG101 for budding (Figure 6B). Remarkably, ectopic expression of Nedd4-2s (Figure 6B, lane 5) suppressed much of the inhibitory effect of DUb-TSG101 on HIV-1 release (Figure 6B, compare lanes 9 and 10, middle). Interestingly, Nedd4-2s expression also restored a detectable measure (~50%) of ubiquitinated Gag that was readily identified in released virus (compare lanes 3 and 5) where higher molecular weight Gag bands (labeled 1, 2 and 3) were also detected with the anti-p24 antibody (lanes 7–10, darker exposure). Conversely, ubiquitination of DUb-TSG101 in cells remained undetectable (Figure 6B, lanes 4 and 5, upper). To ascertain that Gag is the target of ubiquitination at sites of virus budding, we isolated ubiquitinated proteins using agarose beads coated with anti-HA monoclonal antibodies from P100 membrane fraction prepared from transfected cells, which has been shown to be enriched in Gag-containing complexes undergoing assembly into nascent virions [43] and probed for Gag with an anti-p24 antibody. We reasoned that if we can capture ubiquitinated Gag in these fractions, we might be able to detect clear differences in Gag ubiquitination levels in absence or presence of DUb-TSG101 (deubquitination) and upon co-expression with Nedd4-2s (re-ubiquitination). Using an anti-p24 antibody, a robust signal of high molecular weight bands was detected with wt Gag (Figure 6C lane 1, upper) in stark contrast to Gag co-expressed with DUb-TSG101 (lane 2). Loss of ubiquitinated Gag signal at the membrane coincided with strong inhbition of virus release (Figure 6C, third panel from top, compare lanes 1 and 2). Interestingly distinct high molecular weight Gag proteins-- labeled them 1, 2 and 3-- reappeared in the P100 fraction upon ectopic expression of Nedd4-2s (lane 3, upper panel). These bands were also prominently detected in the wt Gag fraction (lane 1) but remained absent from cells expressing DUb-TSG101 in multiple experiments. Differences in detection of ubiquitinated Gag proteins (upper panel) were not due to variability in amounts of Gag captured at the membrane, since P100 fractions exhibited identical levels of p55Gag for all samples (Figure 6C, second panel, lanes 1–3). Importantly, appearance of ubiquitinated Gag at the membrane following co-expression with Nedd4-2 was accompanied with the recovery of about 50% of virus release (Figure 6C, third panel, compare lanes 1 and 3) and a Gag processing pattern that is near indistinguishable from that of wt Gag (no p25-24CA doublet). Only a partial recovery of Gag ubiquitination was seen following ectopic expression of Nedd4-2s. This was expected because Gag at these sites is also associated with DUb-TSG101, which although itself permanently deubiquitnated continues to also deubiquitinate Gag as Nedd4-2s is overcoming such activity. Together these findings indicate that wt Gag is robustly ubiquitinated at the membrane, the site of HIV budding. Inhibition of Gag ubiquitination following co-expression with DUb-TSG101 was accompanied with loss of virus release. Remarkably, a detectable Nedd4-2s-mediated recovery of Gag ubiquitination at the membrane that was qualitatively identical to that of wt Gag (same bands appear in both samples) correlated with restoration of virus release further emphasizing the importance of Gag ubiquitination in HIV budding.

Altogether, the data above suggested that ubiquitination of TSG101 is dispensable for HIV-1 budding and that ubiquitination of the Gag protein itself better correlates with the ability to undergo viral release. As a correlate, overexpression of Nedd4-2s did not restore budding activity to Gag-DUb when expressed alone (Figure 6D, lanes 4–6) nor did Nedd4-2s suppress the inhibitory effect imposed by Gag-DUb on HIV-1 budding (lanes 7–9). As expected, co-expression of Nedd4-2s in these conditions did suppress the budding defect of the HIV-1 PTAP- mutant virus lacking its ability to bind TSG101 (lanes 10–12, PTAP-). Thus, Gag-DUb, which would represent the most powerful and proximal way of eliminating Gag ubiquitination, was resistant to the stimulatory effects of Nedd4-2s co-expression. These data therefore suggest that ubiquitination of Gag is important for HIV-1 release.

**Figure 7:**
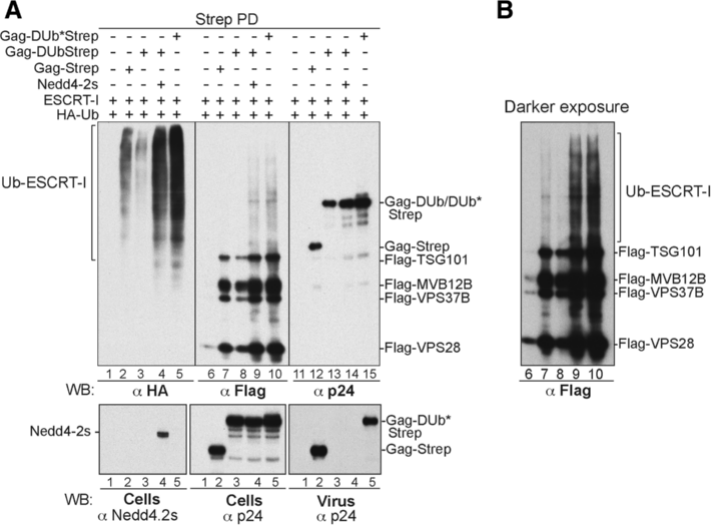
**Ubiquitination of ESCRT-I is not sufficient for HIV-1 release. (A and B)** 293T cells were co-transfected with HA-Ub plasmid and Flag-tagged version of ESCRT-I components [TSG101 (2.5 μg), VPS28 (800 ng), VPS37B (1.7 μg), MVB12B (800 ng); these amounts express comparable levels of ESCRT-I proteins] alone, or in addition to the following plasmids: either Gag-Strep (1.5 μg) (lanes 2, 7, 12) or Gag-DUbStrep alone (1.5 μg) (lanes 3, 8, 13) or in combination with the Nedd4-2s expression plasmid (150 ng) (lanes 4, 9, 14) and with Gag-DUb*Strep alone (1.5 μg) (lanes 5, 10, 15). Sequential centrifugations were performed to separate membrane-enriched P100 fractions from which Gag molecules were immunoprecipitated using *Strep-Tactin* beads. The protein content of captured complexes **(A)**, input (lower left and center panels) and virus (lower right panel) fractions were analyzed by WB with the indicated antibodies. **(B)** shows a darker exposure of samples analyzed in lanes 6–10).

To better ascertain the key target of ubiquitination during viral budding, we examined the ubiquitination status of proteins participating in the defective budding of Gag-DUb. Gag-DUb was affinity captured on *Strep*-tactin beads from cells expressing Flag-tagged ESCRT-I subunits, HA-Ub, and co-expressing Nedd4-2s. Gag-DUb was isolated from a P100 membrane fractions known to be enriched in Gag-containing complexes undergoing assembly into virus-like particles [43]. The captured Gag-containing complexes were immuno-blotted for ESCRT-I components using an anti-Flag antibody (Figure 7A, lanes 6–10), for levels of ubiquitinated proteins using an anti-HA antibody (Figure 7A, lanes 1–5), and for the Gag protein itself using anti-p24 antibodies (Figure 7A, lanes 11–15). All Gag proteins retain the ability to associate with ESCRT-I members regardless of whether Gag was fused to active or inactive DUb forms. However, lower amounts of TSG101 were captured with Gag-DUb in comparison to the WT or the enzymatically inactive Gag-DUb (lanes 7, 8 and 10). In the absence of Nedd4-2s, complexes containing Gag-DUb had greatly diminished levels of ubiquitinated proteins compared with complexes of WT Gag or inactive Gag-DUb*. Importantly, overexpression of Nedd4-2s markedly increased the level of Ub-conjugates in Gag-DUb complexes and enhanced their ability to capture TSG101/ESCRT- complexes (compare lanes 8 and 9). These ubiquitinated proteins correspond to modified ESCRT-I subunits as verified by the high molecular weight smear observed in anti-Flag probed immunoblot for ESCRT-I subunits themselves (lanes 6–10 and panel B showing a darker exposure of this blot). The intensity of these bands also correlate with levels of ubiquitination seen with the anti-HA antibody probed immunoblot (lanes 1–5). No high molecular weight forms of Gag-DUb were observed in the anti-p24 immunoblot as expected since fusion of DUb directly to Gag should constitute the most potent protection of Gag against ubiquitination. Despite restoration of Ub to ESCRT-I associated with Gag-DUb containing assembling particles, Figure 7 (third lower panel on right) shows that release of these particles was not restored. These data indicate that while Gag proteins and ESCRTs both undergo ubiquitination, ubiquitination of ESCRT-I is not sufficient for completion of HIV-budding and release. Rather, the inhibitory effects of Gag-DUb, DUb-TSG101, and DUb-Alix are mediated by blocking ubiquitination of Gag proteins they associate with.

### Incorporation of Gag-Ub into assembly sites alleviates DUb-Alix inhibitory effect

**Figure 8:**
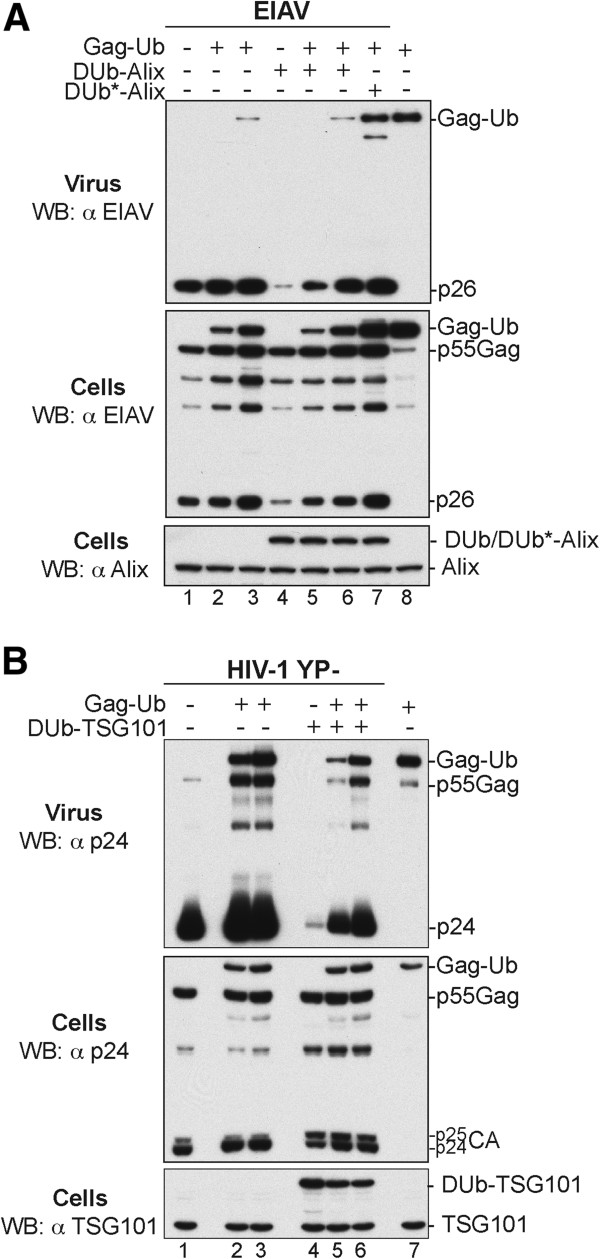
**Incorporation of Gag-Ub into assembly sites alleviates DUb-ESCRT inhibitory effects. A)** 293T cells expressing EIAV (lane 1),were also transfected with increasing amounts of EIAV Gag-Ub expression vector (500 ng and 1μg) (lanes 2 and 3), with Flag-DUb-Alix alone (100 ng) (lane 4), in combination with increasingamounts of Gag-Ub (500 ng and 1μg) (lanes 5 and 6), or with inactive DUb*-Alix alone (100 ng) (lane 7) whereas lane 8 shows expression of Gag-Ub alone. Virions and cells were harvested 24 h post-transfection and their protein contents analyzed by WB using an anti-EIAV antibody. Alix and DUb/DUb*-Alix fusion proteins were detected by an anti-Alix antibody. **B)** 293T cells expressing HIV-1 YP- (lane 1), were also transfected with increasing amounts of HIV Gag-Ub expression vector (1 and 2 μg) (lanes 2 and 3), with DUb-TSG101 alone (200 ng) (lane 4), or in combination with increasing amounts of HIV Gag-Ub expression vector(1 and 2 μg) (lanes 5 and 6) whereas lane 7 shows expression of HIV Gag-Ub alone (1 μg). Virions and cells were harvested 24 h post-transfection and their protein contents analyzed by WB using an anti-p24CA antibody. TSG101 and DUb-TSG101 fusion protein were detected with an anti-TSG101 antibody.

Ectopic expression of DUb-TSG101 or DUb-Alix with HIV and EIAV, respectively, caused severe budding defects. Association of DUb-fusion proteins with Gag led to a clear loss of Gag ubiquitination at virus assembly sites (Figure 7), a defect that was alleviated with ectopic expression of Nedd4-2s and was accompanied with the appearance of ubiquitinated Gag proteins at the membrane. In contrast, fusion of DUb to Gag led to a permanent deubiquitination and an irreversible adverse effect on HIV release despite a robust and detectable ubiquitination of ESCRT-I components. These findings strongly supported a model in which ubiquitin conjugation to Gag is central to ESCRT mediated virus release. To further test this notion, we reasoned that incorporation of ubiquitin molecules to assembly sites in fusion with Gag (Gag-Ub) should recapitulate Nedd4-2s ubiquitination of Gag and restore virus release. To examine this possibility, we utilized EIAV, which relies on the ESCRT machinery to leave the cell and is sensitive to the inhibitory effect of DUb-Alix. In cells expressing EIAV and DUb-Alix in conditions where no virus release was detected, we co-expressed increasing amounts of EIAV Gag-Ub [23] and hypothesized that the latter’s incorporation into Gag assembly sites would rescue virus release if ubiquitin conjugation or presence in the vicinity of Gag is important for ESCRT mediated virus release. Co-expression of Gag-Ub restored robust virus production (Figure 8A, compare lanes 4 and 5) and virus stimulation was proportional to the levels of Gag-Ub expressed *in trans* (lanes 5–7).

To test whether DUb-TSG101 inhibitory effect on HIV budding can be reversed with the incorporation of ubiquitin at Gag assembly sites, we constructed HIV Gag-Ub fusion protein. Remarkably, Alix-independent HIV-1 release became insensitive to DUb-TSG101 inhibitory effect upon co-expression with Gag-Ub (Figure 8B, compare lanes 4 and 5) and virus stimulation was proportional to the levels of Gag-Ub expressed *in trans* (lane 6). Of note, Gag-Ub also enhanced budding of WT virus (compare lane 1 to lanes 2 and 3) further supporting a stimulatory role for Ub conjugation to Gag at assembly sites during HIV exit. Thus incorporation of Gag-Ub into nascent virus relieved DUb-ESCRT inhibitory effects indicating that the mere presence of ubiquitin at Gag assembly sites allowed Gag to bypass DUb-ESCRT-mediated deubiquitination and restored robust virus budding further emphasizing the importance of Gag ubiquitination.

## Discussion

While various studies established that Ub can be sufficient to mediate viral budding, they have not shown that Ub provides a natural and necessary role in the process. The other issue was the lack of efficient tools to directly establish what specific protein(s) in the viral budding process require ubiquitination given that either ubiquitination of the Gag itself or some Gag-binding proteins can be sufficient for virus release. We efficiently deubiquitinated virus budding sites by delivering DUb activity in fusion with Gag or Gag-binding proteins, the ESCRT components TSG101 and Alix. Deubiquitination of virus budding using either type of DUb fusion protein, caused a marked interruption of virus budding as was quantified by both biochemical and electron microscopy analyses. In stark contrast with deubiquitination of ESCRT components, deubiquitination of Gag brought virus release to a complete and irreversible halt despite a measurable ubiquitination of ESCRT components at sites of virus assembly. These data support a central role for Gag ubiquitination in virus budding and provide the first direct demonstration of a critical role for Ub in facilitating this process.

### Ubiquitin is required for virus scission from the cell

The generation of a tool that deubiquitinated virus assembly sites without disruption of function of both viral and cellular proteins involved in virus budding was key to addressing the role of Ub in virus production. Indeed, DUb-TSG101 incorporated in its known cellular complex ESCRT-I and retained sufficient interaction with Gag to be captured at the membrane in late-assembly complexes. Similarly, Alix retained the ability to homodimerize and recruit its ESCRT-III partner CHMP4b. Of note, incorporation of DUb-ESCRTs had no broad adverse effect on the host ESCRT machinery as DUb-TSG101 inhibitory effect was specifically limited to HIV while MoMLV and EIAV, two viruses that also utilize the ESCRT pathway to exit the cell remained insensitive. DUb delivery led to the generation of ubiquitination-resistant assembly sites and arrested nascent viral particles in late steps of budding from the plasma membrane implying that Ub conjugation is involved in virus scission from cells. Interference with virus production required the delivery of an enzymatically active DUb since loss of virus budding was observed only when an active DUb was fused to Gag or ESCRT proteins. As DUb fusion proteins retained their known protein-protein interactions and inhibited virus release only if they retain the ability to incorporate into Gag assembly complexes (Figure 4), it became clear that it is the suppression of Ub conjugation to Gag assembly complexes at the membrane that caused disruption of virus budding and not the mere physical fusion of the DUb catalytic domain to viral or cellular proteins or a broad toxic and debilitating effect on the cell ESCRT pathway. These data revealed a central role for Ub in ESCRT-mediated natural scission of virus from the cell.

### Ubiquitination of ESCRTs is insufficient for virus budding

The mere presence of ubiquitin at sites of virus budding alleviates virus exit dysfunction, whether ubiquitin was conjugated to Gag, ESCRT components or physically fused to Gag [17,23,25-27]. Our data demonstrate that the ubiquitination of Gag itself is critical for virus release. In absence of ESCRT ubiquitination, Gag ubiquitination at sites of budding at the membrane was sufficient to stimulate virus release. Indeed, DUb-TSG101 inhibitory effect on HIV budding was lifted with Nedd4-2s-mediated ubiquitination and virus production correlated with the ubiquitination of Gag complexes at the membrane (Figure 6). Gag deubiquitination however caused an irreversible loss of virus production despite an efficient ubiquitination of TSG101/ESCRT-I at sites of budding, indicating that ESCRTs ubiquitination was insufficient for virus production thus further emphasizing an important role for Gag ubiquitination in this process. It is however important to point out that our findings cannot exclude that ubiquitin-modification of other, yet-to be-identified ESCRT-associated proteins (i.e. proteins that bridge ESCRT-I to ESCRT-III) might be involved in HIV budding. Our data support a model in which both ubiquitin conjugation to Gag and functional L domain sequences are important for HIV budding. Indeed, interference with Gag ubiquitination by cumulative mutations of lysines residues in HIV Gag-- near L domain sequences-- inhibited virus production and arrested budding particles at the membrane [29], although Gag retained an intact L domain. Also, disruption of L domain led to the accumulation of heavily ubiquitinated Gag at the membrane and failure to release virus [31]. Thus deubiquitination of Gag or inability to access ESCRT appears to be equally detrimental to virus budding suggesting both Ub and ESCRT components cooperate in particle budding and separation from cells.

### Ubiquitination of Gag versus ESCRTs

A role for ubiquitin in virus release has been supported by several observations, whether Gag or ESCRT members are the target for such ubiquitination and the extent of their involvement in virus budding remain a matter of debate. We considered both options by generating permanently deubiquitinated DUb fusion proteins of Gag and ESCRTs. Whereas DUb-ESCRT fusion caused an efficient deubiquitination of Gag assembly sites at the membrane and a drastic halt of virus release, this effect was reversible provided Gag was fused to a Ub molecule or enzymatically re-ubiquitinated. Indeed, nascent virus particles that incorporated Gag-Ub molecules escaped the adverse effect DUb-ESCRT imposes on virus release, a phenotype that was recapitulated with Nedd4-2s re-ubiquitination *in trans* of Gag proteins engaged in budding particles at the membrane that were arrested following co-expression of DUb-TSG101. Thus DUb-ESCRT containing complexes appeared to be active (functional) at the membrane at sites of budding as long as Gag gains access to ubiquitin (Figures 6 and 8). The role of ubiquitination of Gag in virus budding is further supported by the findings that incorporation of Gag-Ub fusion protein enhanced WT HIV budding and protected Gag from DUb-TSG101 inhibitory effect of virus production (Figure 8). In contrast, Gag-DUb fusion, which generated ubiquitination resistant virus assembly complexes caused a significant and irreversible loss HIV release and despite a detectable ubiquitination of ESCRT-I associated with nascent virus particles at the membrane (Figure 7). These results do not exclude that ESCRTs or an associated protein is ubiquitinated and that such ubiquitination might play a role in HIV budding, they however reveal that Gag is robustly ubiquitinated at the membrane and that Ub conjugation to Gag plays a prominent and important role for ESCRT-mediated HIV budding.

### Ubiquitination of Gag is central to ESCRT-mediated virus budding

Why Gag requires Ub conjugation to drive virus budding is not clear. Ub appears to be involved but not indispensable for recruitment of ESCRTs since Gag-DUb retained the ability to recruit TSG101/ESCRT-I, albeit with a reduced efficiency (Figure 1D and 7). Accordingly, disruption of TSG101 Ub-binding residues had no detectable effect on TSG101 ability to drive virus release [17]. Ub binding appears to be more important for Alix-mediated HIV budding as loss of Ub binding sites in Alix coincides with inability to function in virus scission from cells [37]. To stimulate HIV budding, Gag displayed a clear preference for Nedd4-2s [17,19]. Interestingly, Nedd4-2s preferentially conjugates K63 ubiquitination [44], a type of ubiquitin modification that is important for cargo trafficking through MVB [45-47] and one that appears to be sufficient for HIV budding [26]. We found that in absence of ESCRTs ubiquitination, Nedd4-2s-mediated ubiquitination of Gag correlated with virus budding (Figures 6 and 7). Remarkably, Nedd4-2s mediated re-ubiquitination of Gag led to the recovery of high molecular weight Gag molecules that were qualitatively identical to those seen with the wt Gag in natural conditions (three distinct bands). Thus HIV Gag seems to share similar requirements with cargo proteins as far as their need for ubiquitin conjugation in ESCRT utilization at sites of membrane scission. Indeed, ubiquitination of the ESCRT apparatus is not required for the sorting of ubiquitinated cargo into the MVB [38]. Thus HIV Gag appears to mimic cargo in its dependence on Ub to process ESCRT-dependent membrane scission and separation from cells [48]. One possible model is that Gag utilizes L domains to capture ESCRT during assembly [49] whereas Ub would be involved in both ESCRT capture as well as at a later stage in the coordination of downstream ESCRT-III function at budding necks. Only a small subset of Gag is ubiquitinated [28,30], which potentially position in budding necks and participate in late–assembly events of membrane scission. Collectively, our studies draw a parallel between the cell cargo proteins and Gag in regard to their functional requirements for budding away from the cytoplasm and provide the first direct demonstration of a natural and necessary role for Gag ubiquitination in ESCRT-mediated virus budding.

## Conclusions

We found that disruption of either L domain sequences or interference with Gag ubiquitination is equally detrimental to virus release, suggesting that although Ub molecules conjugated to Gag are not essential for ESCRT recruitment, Ub conjugation to Gag carrying intact L domains is necessary for Gag utilization of ESCRTs at budding sites to separate virus from cells. We propose a model in which Gag exhibits a dual and strict requirement for both ubiquitin conjugation and L domain recruitment of TSG101 and Alix to promote ESCRT- mediated HIV budding.

## Methods

### Proviral and expression vectors

We used the wild-type molecular clones of HIV-1 pNL4-3 [50] MoMLV [51] and EIAVUK [52]. The HIV-1 L-domain mutants PTAP-, YP- and the PTAP-/YP- were previously described in [42]. Alix full length and the Bro1 (1–367) and VPRD (364–868) fragments were descried in [53] and subcloned into p3XFLAG-myc-CMV-26 vector (Sigma, St. Louis, MO) between the *Not*I/*EcoR*I restriction sites. The Flag-TSG101 expression vector was previously described in [54]. TSG101 cDNA were also subcloned in pEXPR-IBA105 vector (IBA BioTAGnology, IBA US Distribution Center, St. Louis, MO, USA) between *EcoR*I/*Not*I to generate an N-terminally tagged Strep-TSG101. Nedd4.2s lacking the first 121 residues of Nedd4.2 was amplified from the full-length Nedd4.2 [18] and subcloned in pcDNA3 (Invitrogen life technologies, Grand Island, NY) using *Hind*III/*Kpn*I sites. The CHMP4B expression vector was generated by PCR amplification from CHMP4B cDNA (GeneCopoeia, Germantown, MD, USA) and subcloned into pHM6 (Roche, Indianapolis, Indiana, USA) to obtain a N-terminally tagged HA-CHMP4B. The N-terminal residues 15–260 (UL36) of the type I Herpes virus VP1/2 tegument protein [55] was cloned in frame with the 5’ end of Alix and TSG101 cDNA in the p3xFlag-myc-CMV-26 and in pEXPR-IBA105 vector using the *Not*I site [38] to generate DUB-ALIX and DUB-TSG101 fusion proteins. The respective cDNA for VPS28, VPS37 and MVB12 genes were inserted in the p3xFlag-myc-CMV-26. The Rev-independent HIV-1 Gag-Strep construct was amplified by PCR from the HIV-1 Gag-EGFP construct generously provided by Marilyn Resh [56] using a primer reverse contained the Strep tag sequence and cloned in pcDNA3 vector between *BamH*I/*Not*I restriction sites. Additionally, the UL36 and ubiquitin sequence were inserted at the C-terminal of Gag obtaining the Gag-DUBStrep and Gag-Ub fusion protein respectively. The N-terminal HA-tagged Ubiquitin were gifts from Arianna Calistri. To generate GST expression vectors, the HIV-1 NC-p1-p6 and the EIAV NC-p9 coding regions were subcloned in pGEX-5X-2 (GE Healthcare Biosciences, Piscataway, NJ) between *Bam*HI/*Eco*RI sites.

### Virus release analysis

293T cells were maintained and transfected as previously described [18]. Twenty-four hours after transfection, cells and culture media were harvested and their protein content was analyzed using a protocol previously described [57]. HIV-1 proteins were detected using an anti-HIV-1 p24 monoclonal antibody (clone 183-H12-5C) or NEA-9306. MoMLV and EIAV proteins were detected using a goat anti-p30CA antibody and horse anti-EIAV serum [58], respectively. Other proteins were detected using anti-HA, anti-Flag or anti-tubulin monoclonal antibodies (Sigma, St. Louis, MO). EIAV release ratio (values inpercentage) was calculated using the following: release ratio = virus-associated Gag/ cell associated Gag, as determined by densitometry analysis of Western blot films using ImageJ software (W. S. Rasband, NIH, Bethesda, MD; http://rsb.info.nih.gov/ij). Alix and TSG101 proteins were detected using a polyclonal anti-Alix antibody and a mouse anti-TSG101 antibody (BD Biosciences, San Jose, CA).

### Infectivity assay

Viral infectivity was quantified using TZM-bl cells assay [59] as descried in [60]. Briefly, HeLa TZM-bl cells were seeded (2×10^4^ cells) in 96-well plates and the following day infected in triplicate with HIV-1 YP- or HIV-1 PTAP- rescued virus stocks in presence of 20 g/ml DEAE-dextran (Sigma, St. Louis, MO). After 48 hours, cells were assayed for luciferase activity using the Steady-Glo™ Reagent kit (Promega, Madison, WI) according to manufacturer’s instructions.

### Immunoprecipitation assays

These assays were conducted as previously described [57,60]. Immunoprecipitation complexes and cell lysates (input fractions) were analyzed by SDS-PAGE and western blot using anti-HA, anti-Flag M2 (Sigma, St. Louis, MO).

### GST pull down assays

The empty pGEX vector or that carrying the encoding sequences of NC-p6 and NC were expressed in BL21(DE3) pLysS *E. coli* (Stratagene) and their interaction with Flag-Alix or Flag-DubsAlix were examined in GST-pull down assays following the protocol previously described [57]. Eluate complexes and cell lysates (input fractions) were analyzed by SDS-PAGE and western blot using the indicated antibodies.

### RNAi knockdown

293T cells (2.5 × 106 cells/ml) were transfected with 250 pmol of a mixture of two RNAi oligonucleotides or with 75 pmol of an RNAi oligonucleotide against cellular Alix and cellular TSG101 respectively (Invitrogen life technologies, Grand Island, NY). After 36 h, cells were cotransfected with the same amount of RNAi and 500 ng of EIAV_UK_ proviral DNA and 150 ng of Flag-Alix or Flag-AlixDUB plasmids RNAi resistant (RR) or with 1 μg of HIV-1 YP- proviral DNA and 50 ng of Flag-TSG101 or Flag-TSG101DUB contracts RNAi resistant. Cells and virus were harvested and processed as described above.

### Cells fractionation

Forty-eight hours post transfection, 293T cells were harvested and washed twice with cold PBS. The cells were resuspended in cold Hypotonic Buffer (10 mM Tris pH 7.5, 1 mM MgCl_2_) and kept on ice for 30 min. Cells were broken to release nuclei using a pre-chilled 7 ml Dounce homogenizer. The samples were centrifuged at 1,000 × *g*, 4°C for 15 min to pellet nuclei and the supernatant representing the cytoplasmic fraction was centrifuged at 100,000 × *g* at 4°C for 1 hour to collect the membrane fraction. The pellet was solubilized in RIPA Buffer (0.5% IGEPAL, 50 mM HEPES [pH 7.3], 150 mM NaCl, 2 mM EDTA, 20 mM β-glycerophosphate, 0.1 mM Na_3_VO_4_, 1 mM NaF, 1 mM phenylmethylsulfonyl fluoride, 0.5 mM dithiothreitol, and Complete protease inhibitor cocktail) and used to perform immunoprecipitation assays.

### Electron microscopy

293T cells were seeded at 6×10^5^/well of a 6-well plate and transfected the following day with 2 μg of HIV-1 YP- mutant and DUB-TSG101 expression vectors or with EIAV_uk_ provirus and DUB-Alix plasmids. At 36 h post-transfection, the supernatants were removed and the cells were fixed for 15 min at room temperature in 2% (v/v) glutaraldehyde in 0.1 M cacodylate buffer (pH 7.4). The cells were then rinsed in cacodylate buffer and postfixed in 1% (v/v) osmium tetroxide in the same buffer. The samples were subsequently rinsed again in 0.1 N sodium acetate buffer (pH 4.2), stained in 0.5% uranyl acetate (v/v) in the same buffer, dehydrated in graded ethanol, then infiltrated overnight in pure epoxy resin. The wells were embedded in fresh resin the next day and cured at 55°C. Blocks were cut from the cured samples and mounted appropriately for ultramicrotomy. Thin sections were stained in uranyl acetate and lead citrate and stabilized by carbon evaporation. Images were obtained with a Hitachi H7600 electron microscope equipped with an AMT XL41M digital camera. Approximately 600 cells were examined for each sample and arrested budding structures attached to the cell as well as released virions were enumerated to determine the release efficiency.

## Competing interests

The authors declare that they have no competing interests.

## Authors’ contributions

PS co-designed, performed all experiments in the paper except electron microscopy, analyzed data and co-wrote the paper. KN performed electron microscopy experiments. RP provided DUb constructs and co-wrote the paper. FB designed experiments, analyzed the data and wrote the paper. All authors read and approved the final manuscript.

## Supplementary Material

Additional file 1: Figure S1 Effect of DUb-TSG101 in HIV-1 and MoMLV release. **(A)** DUb-TSG101 interferes with HIV-1 release. 293T cells were transfected with expression plasmids of HIV-1 (lane 1) and Flag-TSG101, Flag-DUb-TSG101 or Flag-DUb*-TSG101 expression plasmids (lanes 2, 3, 4, respectively). **(B)** DUb-TSG101 had no effect on MoMLV release. 293T cells were transfected with MoMLV proviral DNA alone (lane 1), with Flag-DUb-TSG101 (lane 2) or Flag-DUb*-TSG101 (lane 3). **(C)** EIAV budding is immune to DUb-TSG101 inhibitory effects. 293T cells were transfected with EIAV proviral DNA alone (lane 1), with Flag-DUb-TSG101 (lane 2) or Flag-DUb*-TSG101 (lane 3). **(D)** DUb-TSG101 M95A mutant fails to inhibit HIV-1 release: 293T cells were transfected with expression plasmids of HIV-1 YP- alone (lane 1), or with Flag- TSG101 (lane 2), DUb-TSG101 (lane 3), DUb-TSG101 M95A mutant (lane 4) or the inactive form DUb*-TSG101 (lane 5). Cells and viruses were collected 24 hours post- transfection and their protein content was analyzed by WB blot using the indicated antibodies. Virus release was quantified from 3 independent experiments and expressed relative to WT virus. **Figure S2:** DUb-Alix and DUb*-Alix fusion proteins retain ability to dimerize. 293T cells were transfected with HA-tagged Alix, DUb-Alix or DUb*-Alix expression vectors alone (First panel, lanes 1, 5 and 9) or in combination with Flag-Alix (lanes 2, 6 and 10), Flag-V-PRD (lanes 3, 7 and 11) or Flag-Bro1 (lanes 4, 8 and 12). HA-tagged proteins were immunoprecipitated on HA-antibody conjugated beads and captured protein complexes (Second panel) were probed for their ability to interact with Flag-Alix and Alix fragments V-PRD and Bro1. Protein complexes and cell lysates (input fractions) were analyzed by WB blot using the indicated antibodies. **Figure S3** DUb-Alix has no detectable effect on HIV-1 production. 293T cells were transfected with HIV-1 provirus DNA alone (lane 1), or in combination with either the Flag-DUb-Alix (lane 2) or DUb*-Alix (lane 3). Samples were processed as described in **Figure S1**. Click here for file
