# Supplementary material for: Ubiquitin conjugation to Gag is essential for ESCRT-mediated HIV-1 budding
Source: Retrovirology. 2013 Jul 29;10:79. doi: 10.1186/1742-4690-10-79 (PMC3751857; doi:10.1186/1742-4690-10-79)
Supplement: Additional file 1: Figure S1 — Effect of DUb-TSG101 in HIV-1 and MoMLV release. (A) DUb-TSG101 interferes with HIV-1 release. 293T cells were transfected with expression plasmids of HIV-1 (lane 1) and Flag-TSG101, Flag-DUb-TSG101 or Flag-DUb*-TSG101 expression plasmids (lanes 2, 3, 4, respectively). (B) DUb-TSG101 had no effect on MoMLV release. 293T cells were transfected with MoMLV proviral DNA alone (lane 1), with Flag-DUb-TSG101 (lane 2) or Flag-DUb*-TSG101 (lane 3). (C) EIAV budding is immune to DUb-TSG101 inhibitory effects. 293T cells were transfected with EIAV proviral DNA alone (lane 1), with Flag-DUb-TSG101 (lane 2) or Flag-DUb*-TSG101 (lane 3). (D) DUb-TSG101 M95A mutant fails to inhibit HIV-1 release: 293T cells were transfected with expression plasmids of HIV-1 YP- alone (lane 1), or with Flag- TSG101 (lane 2), DUb-TSG101 (lane 3), DUb-TSG101 M95A mutant (lane 4) or the inactive form DUb*-TSG101 (lane 5). Cells and viruses were collected 24 hours post- transfection and their protein content was analyzed by WB blot using the indicated antibodies. Virus release was quantified from 3 independent experiments and expressed relative to WT virus. Figure S2: DUb-Alix and DUb*-Alix fusion proteins retain ability to dimerize. 293T cells were transfected with HA-tagged Alix, DUb-Alix or DUb*-Alix expression vectors alone (First panel, lanes 1, 5 and 9) or in combination with Flag-Alix (lanes 2, 6 and 10), Flag-V-PRD (lanes 3, 7 and 11) or Flag-Bro1 (lanes 4, 8 and 12). HA-tagged proteins were immunoprecipitated on HA-antibody conjugated beads and captured protein complexes (Second panel) were probed for their ability to interact with Flag-Alix and Alix fragments V-PRD and Bro1. Protein complexes and cell lysates (input fractions) were analyzed by WB blot using the indicated antibodies. Figure S3 DUb-Alix has no detectable effect on HIV-1 production. 293T cells were transfected with HIV-1 provirus DNA alone (lane 1), or in combination with either the Flag-DUb-Alix (lane 2) or DUb*-Alix (lane 3). [file 1742-4690-10-79-S1.pdf]

Supplemental figure legends:

Fig. S1: Effect of DUb-TSG101 in HIV-1 and MoMLV release. (A) DUb-TSG101 interferes with HIV-1 release. 293T cells were transfected with expression plasmids of HIV-1 (lane 1) and Flag-TSG101, Flag-DUb-TSG101 or Flag-DUb*-TSG101 expression plasmids (lanes 2, 3, 4, respectively). **(B) DUb-TSG101 had no effect on MoMLV release.** 293T cells were transfected with MoMLV proviral DNA alone (lane 1), with Flag-DUb-TSG101 (lane 2) or Flag-DUb*-TSG101 (lane 3). **(C) EIAV budding is immune to DUb-TSG101 inhibitory effects.** 293T cells were transfected with EIAV proviral DNA alone (lane 1), with Flag-DUb-TSG101 (lane 2) or Flag-DUb*-TSG101 (lane 3). **(D) DUb-TSG101 M95A mutant fails to inhibit HIV-1 release:** 293T cells were transfected with expression plasmids of HIV-1 YP- alone (lane 1), or with Flag-TSG101 (lane 2), DUb-TSG101 (lane 3), DUb-TSG101 M95A mutant (lane 4) or the inactive form DUb*-TSG101 (lane 5). Cells and viruses were collected 24 hours post-transfection and their protein content was analyzed by WB blot using the indicated antibodies. Virus release was quantified from 3 independent experiments and expressed relative to WT virus.

Fig. S2: DUb-Alix and DUb*-Alix fusion proteins retain ability to dimerize. 293T cells were transfected with HA-tagged Alix, DUb-Alix or DUb*-Alix expression vectors alone (First panel, lanes 1, 5 and 9) or in combination with Flag-Alix (lanes 2, 6 and 10), Flag-V-PRD (lanes 3, 7 and 11) or Flag-Bro1 (lanes 4, 8 and 12). HA-tagged proteins were immunoprecipitated on HA-antibody conjugated beads and captured protein complexes (Second panel) were probed for their ability to interact with Flag-Alix and Alix fragments V-PRD and Bro1. Protein complexes and cell lysates (input fractions) were analyzed by WB blot using the indicated antibodies.

Fig. S3 DUb-Alix has no detectable effect on HIV-1 production. 293T cells were transfected with HIV-1 provirus DNA alone (lane 1), or in combination with either the Flag-DUb-Alix (lane 2) or DUb*-Alix (lane 3). Twenty-four hours later, cells and virus were harvested, viral particles pelleted and their protein content analyzed by WB blot

using the indicated antibodies. Relative virus release efficiencies were calculated from three independent experiments and expressed relative to the WT provirus and shown under panels. Error bars represent standard deviations (SD).

S1

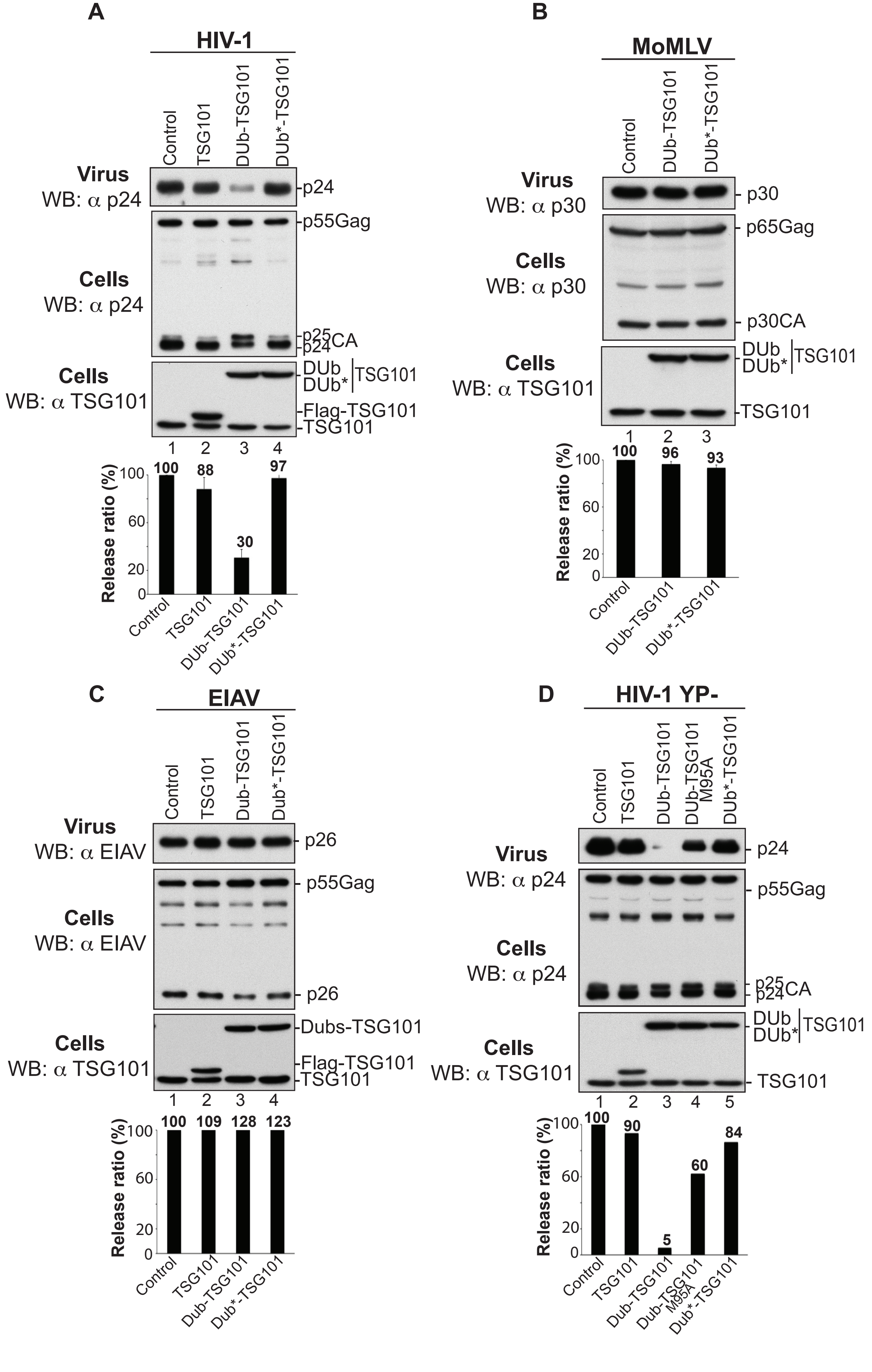

A
HIV-1
Control
TSG101
DUb-TSG101
DUb*-TSG101
Virus
WB: α p24
p24
p55Gag
Cells
WB: α p24
p25
p24
CA
Cells
WB: α TSG101
DUb
DUb*
TSG101
Flag-TSG101
TSG101
1 2 3 4
Release ratio (%)
100 88 30 97
B
MoMLV
Control
DUb-TSG101
DUb*-TSG101
Virus
WB: α p30
p30
p65Gag
Cells
WB: α p30
p30CA
Cells
WB: α TSG101
DUb
DUb*
TSG101
TSG101
1 2 3
Release ratio (%)
100 96 93
C
EIAV
Control
TSG101
Dub-TSG101
Dub*-TSG101
Virus
WB: α EIAV
p26
p55Gag
Cells
WB: α EIAV
p26
Cells
WB: α TSG101
Dubs-TSG101
Flag-TSG101
TSG101
1 2 3 4
Release ratio (%)
100 109 128 123
D
HIV-1 YP-
Control
TSG101
DUb-TSG101
DUb-TSG101 M95A
DUb*-TSG101
Virus
WB: α p24
p24
p55Gag
Cells
WB: α p24
p25
p24
CA
Cells
WB: α TSG101
DUb
DUb*
TSG101
TSG101
1 2 3 4 5
Release ratio (%)
100 90 5 60 84
Dub-TSG101 M95A
Dub*-TSG101

S2

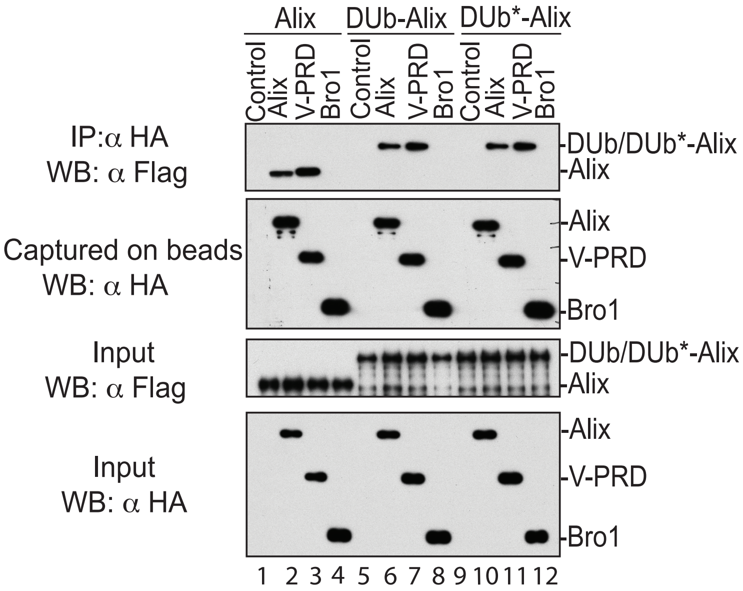
Alix
DUb-Alix
DUb*-Alix
Control
Alix
V-PRD
Bro1
Control
Alix
V-PRD
Bro1
Control
Alix
V-PRD
Bro1
IP:α HA
WB: α Flag
-DUb/DUb*-Alix
-Alix
Captured on beads
WB: α HA
-Alix
-V-PRD
-Bro1
Input
WB: α Flag
-DUb/DUb*-Alix
-Alix
Input
WB: α HA
-Alix
-V-PRD
-Bro1
1 2 3 4 5 6 7 8 9 10 11 12

S3

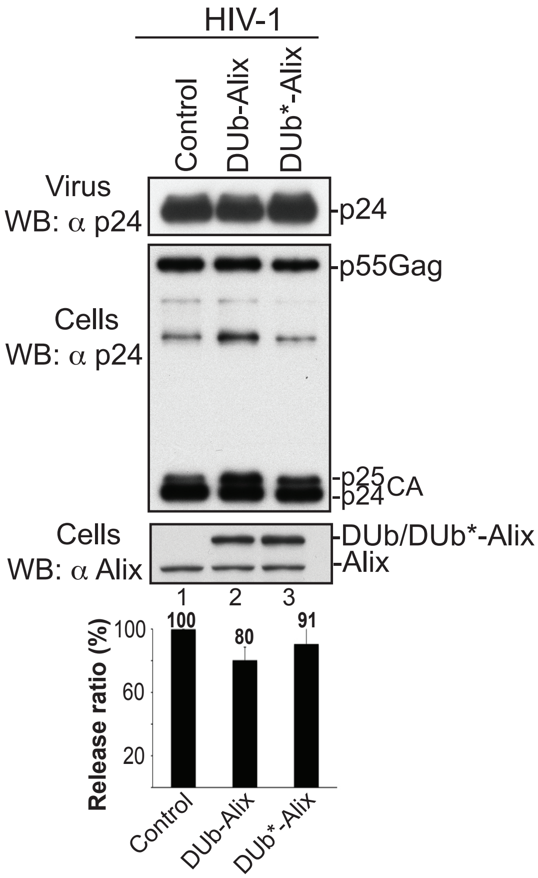
HIV-1
Control
DUb-Alix
DUb*-Alix
Virus
WB: α p24
-p24
-p55Gag
Cells
WB: α p24
-p25
-p24
CA
Cells
WB: α Alix
-DUb/DUb*-Alix
-Alix
1
2
3
Release ratio (%)
100
60
20
100
80
91
Control
DUb-Alix
DUb*-Alix
